# An H-*TERT* Mutated Skin Metastasis as First Occurrence in a Case of Follicular Thyroid Carcinoma

**DOI:** 10.3389/fendo.2019.00513

**Published:** 2019-07-31

**Authors:** Eleonora Monti, Mariella Dono, Edoardo Gonella, Bruno Spina, Francesca Pitto, Floriana Petrogalli, Lucia Conte, Eleonora Ambrosetti, Michele N. Minuto, Gian Luca Ansaldo, Silvia Morbelli, Simona Zupo, Massimo Giusti

**Affiliations:** ^1^Endocrinology Unit, Department of Internal Medicine (DI.M.I.), IRCCS Ospedale Policlinico San Martino, University of Genoa, Genoa, Italy; ^2^Molecular Diagnostic Unit, IRCCS Ospedale Policlinico San Martino, Genoa, Italy; ^3^Department of Pathology, IRCCS Ospedale Policlinico San Martino, Genoa, Italy; ^4^Department of Pathology, ASL3 Genovese, Genoa, Italy; ^5^Department of Surgical Sciences (DISC), UO Chirurgia 1 IRCCS Ospedale Policlinico San Martino, University of Genoa, Genoa, Italy; ^6^Department of Nuclear Medicine, IRCCS Ospedale Policlinico San Martino, Genoa, Italy

**Keywords:** follicular thyroid carcinoma, skin metastasis, indeterminate lesions, THY 3, *TERT* promoter, *NRAS*

## Abstract

Differentiated thyroid cancer arising from thyroid follicular epithelial cells is the most frequent endocrine malignancy, and skin metastases are very rare. We describe a case of a 70-year-old women with a history of an indeterminate thyroid nodule on cytology. A painless, erythematous skin nodule of about 7 mm diameter was removed from the scalp and diagnosed as a metastasis from thyroid cancer. After total thyroidectomy, a histological diagnosis of follicular thyroid cancer was made. Two cycles of radioactive iodine were performed. Both the follicular thyroid carcinoma (FTC) and the metastasis were investigated for the presence of *BRAF/RAS* and *TERT* promoter mutations. The results showed that the cutaneous metastasis was *BRAF* wild-type and *TERT* promoter-mutated (position g.1,295,228 C>T); in contrast, the primary thyroid lesion was negative for both molecular markers.

## Introduction

The most frequent endocrine malignancy is differentiated thyroid carcinoma (DTC). Regional cervical lymph nodes are the most common sites of local metastases, while lung and bone are the most usual sites of distant metastases. Skin metastases are very rare, the scalp being the most frequent site (1). We report the case of a woman who presented a single skin lesion as the first sign of DTC, in which the metastatic site, but not the primary tissue, displayed *TERT* mutation.

## Case Report

**Figure 1:**
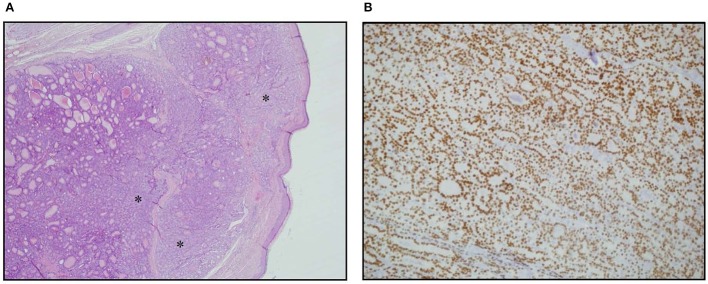
Skin metastasis. **(A)** Hematoxylin Eosin staining of histological slides, magnification 4 × 1. *indicates less differentiated areas. **(B)** Immunohistochemical staining of TTF-1, magnification 20 × 1.

**Figure 2:**
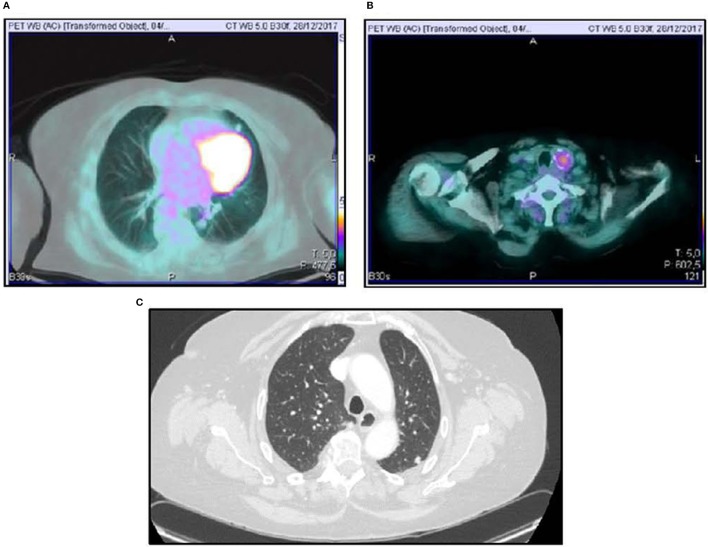
18FDG-PET scan showing lung uptake **(A)** and thyroid uptake **(B)**. Computed Tomography Imaging of lung metastasis **(C)**.

A 70-year-old women was referred to an endocrinology center owing to multinodular goiter. She had a family history of benign nodular thyroid pathology. Thyroid ultrasound revealed a multinodular goiter with a 20 mm hypoechoic nodule in the right lobe and a 25 mm hypoechoic nodule with microcalcifications in the left lobe. Her thyroid function was unknown, but oral levo-thyroxine therapy was started in November 2014. Fine-needle cytology (FNAC) on the right nodule confirmed the benign nature of the lesion: THY 2 according to the second edition of the British Thyroid Association (BTA) classification (2). FNAC on the left nodule showed an indeterminate lesion (THY 3 according to BTA 2007); 3 months later, a new cytological evaluation was performed. This second FNAC confirmed the indeterminate cytology (THY 3 A according to the third BTA classification, BTA 2014) (3). Analysis of *BRAF* V600E mutation of the cytological specimens revealed a wild-type genotype. Thus, the patient was advised to undergo ultrasound follow-up, but refused. After 2 years, the patient noticed a painless, erythematous skin nodule of about 7 mm diameter on the scalp. The lesion was removed and the histological diagnosis suggested a thyroidal origin; the nodule was composed of epithelial cells arranged in follicular structures which contained amorphous material resembling thyroid colloid (Figure 1A). On immunohistochemical staining, neoplastic cells showed expression of TTF-1 (Figure 1B), Cytokeratin 7, and Thyroglobulin (Tg); thus, a diagnosis of probable metastasis from a follicular thyroid carcinoma (FTC) was made. When the patient was referred to our clinic (November 2017), her BMI was high (43 kg/m2), her thyroid function was normal (TSH 2.33 mIU/L) with high levels of Tg (6,752 μg/L; normal range 3.5–77 μg/L), calcitonin was <1.0 ng/L and autoimmunity was negative. A thyroid ultrasound scan (US) revealed a hypoechoic right nodule, with microcalcifications of 20 mm, and a hypoechoic left nodule with microcalcifications of 50 mm. After collegial discussion with the surgeon, nuclear medicine specialist and pathologist, a total-body PET 18F-FDG scan (with a Siemens somatom sensation 16 biograph, Rome, Italy) was performed; this showed a lung metastasis with poor 18-FDG uptake (Figure 2A) and a left thyroid nodule with moderate 18-FDG uptake (Figure 2B).

Total thyroidectomy with central compartment neck dissection was performed, followed by thyroid ablation with 120 mCi of radioiodine (RAI). To prepare for the ablation, the patient underwent recombinant human (rh) TSH stimulation (Thyrogen, four i.m. injections of 1.8 mg to the buttock in the 4 days preceding radioiodine administration). Although total hormone withdrawal would be the recommended choice, the use of rhTSH was justified by the patient's psychological condition, and the clinicians consented to this therapy (4). Indeed, a number of literature reports have indicated that both treatments may have the same type of effect (5, 6). However, the second course of radioiodine therapy was administered under total hormone withdrawal, since the patient had, by that time, acquired enough confidence in the physicians' advice. After 131I administration, a whole-body x-ray scan was performed by means of a Philips Forte (Rome, Italy) gamma camera, and identified some areas of pathological hyperaccumulation of the tracer: lungs, one vertebra and right hypochondrium. We then performed a total-body CT scan, which revealed a liver metastasis and confirmed the lung metastases (Figure 2C).

**Figure 3:**
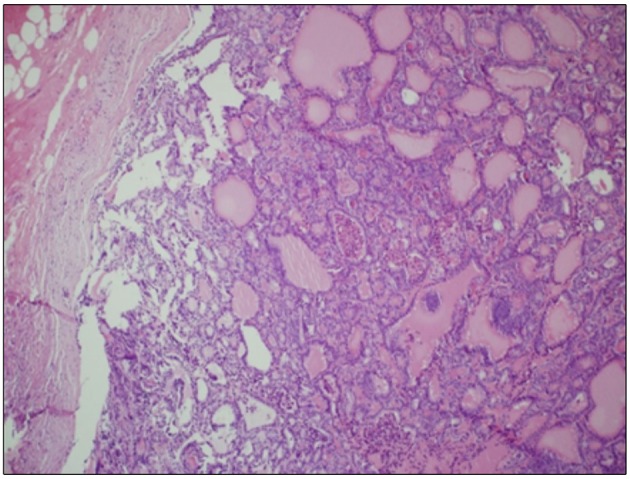
Thyroid tissue. Hematoxylin Eosin staining of histological slides, magnification 10 × 1.

Histological examinations of thyroid tissue showed a macroscopic 45 mm nodule on the left lobe with the morphological features of a follicular cancer, and both capsular and vascular invasion, thus resembling a “widely invasive” FTC (WHO classification, 2017) in the context of diffuse nodular hyperplasia (Figure 3 and Supplementary Figure S1).

The second course of RAI treatment, which was administered at 250 mCi under thyroid hormone withdrawal, led to a reduction in iodine uptake by distant metastases. Before this second treatment, the patient's TSH level was 100 U/L, stimulated Tg was 1,037 μg/L and Tg antibodies were negative. At present, neck US is negative for disease recurrence. Before the second ablation, unstimulated Tg was 250 μg/L; it is now 123 μg/L. TSH is suppressed (0.66 mIU/L) on LT4 therapy (875 μg weekly).

**Figure 4:**
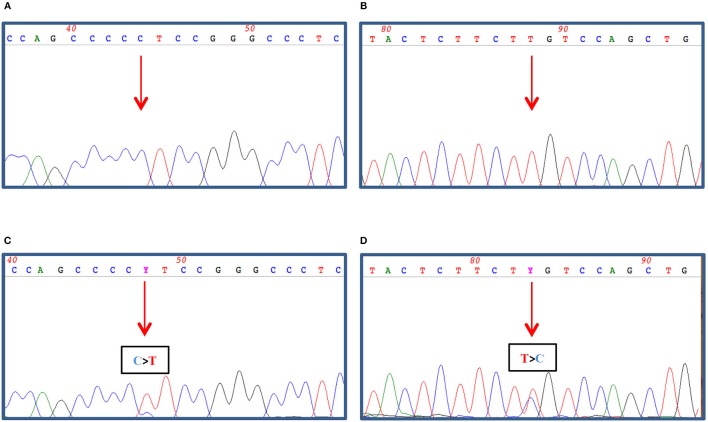
100–200 ng of genomic DNA was extracted from FFPE thyroid tissues **(A,B)** and cutaneous metastasis **(C,D)** by using QIAamp DNA FFPE Tissue Kit (Qiagen, Hilden, Germany) and subjected to *TERT* promoter amplification **(A,C)** by PCR as described in Dono et al. (7) and to *NRAS* exon 3 amplification **(B,D)** by PCR using 1.5 U Platinum Taq DNA polymerase (Thermo Fisher Scientific, Milan, Italy), 1x buffer, 2 mM MgCl2, 200 nM dNTPs, and 30 pmoles of Forward (5′gattcttacagaaaacaagtgg) and Reverse (5′ taatgctcctagtacctagtgag) primers in a final volume of 50 μl. The amplified PCR products were then treated with ExoSap (GeHealthcare, Waukesha, USA) as recommended and both strands sequenced by dye terminator cycle sequencing (BigDye Terminator v3.1, Thermo Fisher). Nucleotide sequence detection was performed on an ABI Prism 3,500 Genetic Analyzer (Thermo Fisher) according to standard protocols. The sequence data were obtained by Mac Vector software analysis (MacVector Inc., North Carolina, USA).

Both the FTC tissue and the skin metastasis were investigated for the presence of *BRAF/RAS* and *TERT* promoter mutations. While both tissues proved to be *BRAF* wild-type, the cutaneous metastasis was *TERT* promoter-mutated (g.1,295,228 C>T) (not shown and Figure 4C); in contrast, the primary thyroid lesion was negative for both molecular markers (not shown and Figure 4A). Interestingly, a NRAS Gln61Arg mutation co-occurring with the *TERT* mutation was found in the cutaneous metastasis (Figure 4D) but not in the primary carcinoma (Figure 4B).

## Discussion and Conclusions

DTC have a good prognosis and >10% of patients develop distant metastases (8). The Italian consensus on thyroid cancer in Thy 3a lesions suggests that conservative management be considered in the case of favorable clinical and US criteria, while in Thy 3f lesions surgery is recommended in most cases (9). The approach to indeterminate nodules is still debated. It is not clear whether the subdivision of Thy 3 lesions into Thy 3a (atypical features) and Thy 3f (follicular lesion) indicates a different oncologic risk, as suggested in the latest version of the BTA classification^3^. Alexander et al. did not observe a real difference in risk, their rates of malignancy being 24.7.7% in Thy 3, 30.4% in Thy 3a, and 29.2% in Thy 3f (10). However, the incidence of thyroid malignancy varied considerably among the four centers involved in their study (Thy 3f 18–54%). Moreover, in our experience, no statistically significant difference in the risk of malignancy was found between Thy 3a (26%) and Thy 3f (14%) nodules (11).

In the literature, some authors have found that the most common histological type among patients with skin metastases is FTC (1, 12, 13). In another study, however, Papillary thyroid carcinoma (PTC) was the most frequent tumor type (41%) in patients with skin metastases (14). Moreover, on analyzing 16 patients with skin metastases, Erickson et al. found 11 PTC and 5 FTC (15). Indeed, more recently, Cohen et al. observed a 1/1,000 incidence of skin metastases in PTC (16).

The most common metastatic site is consensually the scalp, as in our case (1, 12–14). Skin metastasis is rarely the first manifestation of the tumor, as it was in our patient. In fact, Somoza et al. reported a similar case, that of a 71-year-old Caucasian male with a skin metastasis of about 1 cm from a follicular variant of PTC (17), and Smit et al. found a 79-year-old Caucasian woman with skin metastases as the first manifestation of a follicular variant of PTC (18). Jehangir et al. also observed a Caucasian woman with a history of thyroid nodule who had rapidly growing skull nodules and a histological diagnosis of FTC (19), and Shamim et al. published two case reports in which a skull lesion was the first manifestation (20). In all these cases, however, the skin lesions were far more advanced than in our case.

Two recurrent non-coding mutations within the h*TERT* promoter region (chr5:1,295,228 G>A and 1,295,250 G>A) were first described in both familial and sporadic melanomas and later identified in other tumor types, such as glioblastoma, bladder and thyroid cancer, as reviewed in reference (21). As these mutations are present within the promoter, they create new binding sites for the Etwenty-six (ETS) transcription factor family, thus constituting an alternative mechanism of genetic activation in cancer. Indeed, mutated *TERT* causes inhibition of physiological telomere shortening, thereby promoting the immortalization of cancer cells. In thyroid cancer, h*TERT* mutations are usually present in follicular-derived thyroid carcinoma and are associated with more aggressiveness (and advanced forms of disease) (22). In our case, the patient's cutaneous metastasis showed the presence of two concurrent mutations: the 1,295,228 G>A in the *TERT* promoter and the Gln61Arg in the *NRAS* gene. Since expression of members of the ETS family may be induced by *BRAF* and *RAS* mutations, further acquisition of a *TERT* mutation may extend the lifespan of the *BRAF/RAS*-driven clone, resulting in the development of a more advanced disease (23). In our case, both *TERT* and *NRAS* mutations were apparently absent from the primary tumor. However, we cannot exclude that a minor “aggressive” mutated clone, undetectable by means of the standard genotyping techniques, might have already been present within the bulk of the primary tumor and that this clone might have played a role in its progression. This hypothesis seems to be supported by many studies in which distant metastases have been correlated with the presence of a *TERT* promoter mutation, not only in tissues but also when found in thyroid cytology specimens (24, 25). Interestingly, the co-occurrence of *TERT* promoter mutation with *BRAF* alteration is already a strong indicator of the aggressive behavior of disease, as reviewed in the meta-analysis by Jin et al. (26). Indeed, the co-existence of *TERT* promoter and *RAS* mutations in DTC is already known, and the synergistic effect of these co-occurring alterations, in terms of tumorigenesis and aggressiveness, has been described in several studies (27–29). However, the biological and clinical significance of this association needs to be further investigated. In our case, the fact that both mutations were present in the distant metastasis corroborates the idea that they both contributed to the aggressiveness of the tumor. Indeed, in comparison with the primary tumor, the skin metastasis displayed some areas with less differentiation, a finding that may correlate with the appearance of *TERT* and *NRAS* mutations (see Figure 3). Of course, it is difficult to establish which of the *TERT* and *NRAS* mutations occurred earlier, although the hypothesis that the *TERT* mutation was a later occurrence is more likely to explain the progression of the tumor.

In the literature, patients with skull metastases have a worse prognosis. In our case, early treatment with two doses of RAI may have made the prognosis more favorable. *TERT* promoter mutation, as shown in the literature, is a predictor of aggressiveness and poorer prognosis (28–30). At the present time, the patient is still alive, in good condition and totally asymptomatic. In the meantime, she also underwent a third course of RAI treatment; her distant metastases have remained stable (131 Iodine whole-body scan, not shown) and her unstimulated Tg levels were 277 μg/L.

In the case described, adequate imaging follow-up, as suggested by Pacini et al. (9), enabled an early diagnosis to be made and prompt treatment to be undertaken. RAI therapy remains the most efficacious post-surgical treatment, as shown in our case by the reduction of distant metastases and thyroglobulin after the second course of treatment.

## Data Availability

The raw data supporting the conclusions of this manuscript will be made available by the authors, without undue reservation, to any qualified researcher.

## Ethics Statement

Written informed consent was obtained from the individual(s) for the publication of any potentially identifiable images or data included in this article.

## Author Contributions

EM and MG: case review. MD: experimental design and writing. MM and GA: clinical data. BS, FPi, and FPe: pathology data. EG: experimental results. SM: imaging. LC and EA: writing. SZ: review of manuscript. All authors contributed to manuscript revision, read, and approved the submitted version.

### Conflict of Interest Statement

The authors declare that the research was conducted in the absence of any commercial or financial relationships that could be construed as a potential conflict of interest.
