# Supplementary figures and images for: An H-TERT Mutated Skin Metastasis as First Occurrence in a Case of Follicular Thyroid Carcinoma
Source: Front Endocrinol (Lausanne). 2019 Jul 31;10:513. doi: 10.3389/fendo.2019.00513 (PMC6684754; doi:10.3389/fendo.2019.00513)

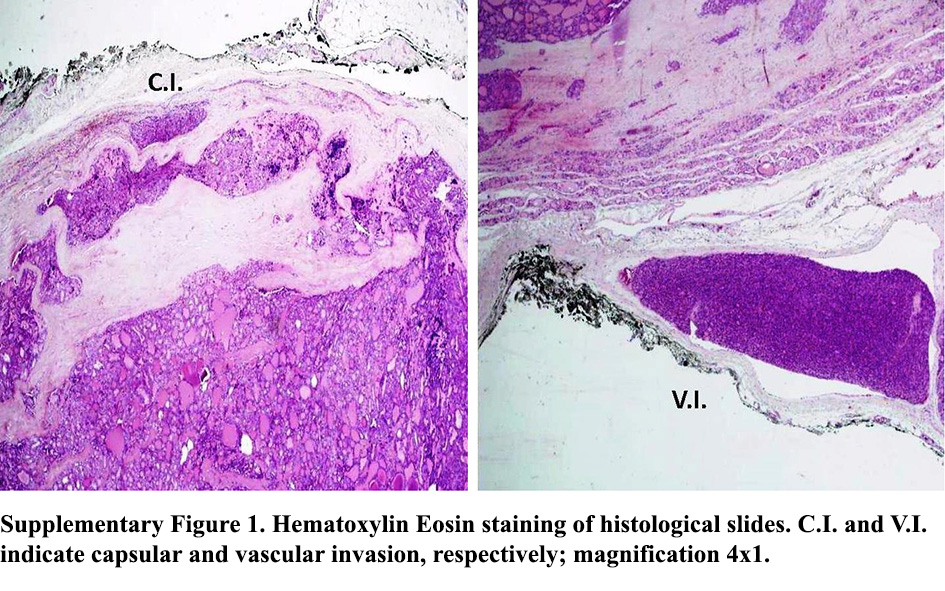

Supplement: Supplementary file 1 [file Image_1.JPEG]
